# Distal Radius Fracture Therapy Utilization Following Traditional Open Reduction and Internal Fixation and Dorsal Bridge Plate Fixation

**DOI:** 10.7759/cureus.54875

**Published:** 2024-02-25

**Authors:** Lilah Fones, Lauren C O'Mara, Gregory Gallant, Moody Kwok, Jack Abboudi, Pedro Beredjiklian

**Affiliations:** 1 Department of Orthopaedic Surgery, Thomas Jefferson University Hospital, Philadelphia, USA; 2 Division of Hand Surgery, Rothman Orthopaedic Institute, Philadelphia, USA

**Keywords:** hand therapy cost, open reduction internal fixation, hand therapy, dorsal spanning plate, dorsal bridge plate, distal radius fracture

## Abstract

Background

Many distal radius fractures are treated with a volar locking plate, but a minority undergo dorsal bridge plate fixation. This study's primary purpose was to compare therapy utilization following distal radius fractures treated with traditional open reduction and internal fixation (ORIF) versus dorsal bridge plate fixation. Secondary outcomes were time to first and last therapy visits and therapy costs.

Methods

Patients over 18 years old who underwent distal radius ORIF between January 2021 and August 2022 at a single regional orthopedic practice were identified. Patients who underwent post-operative hardware removal were retrospectively reviewed to identify dorsal bridge plate fixation patients. This resulted in "traditional ORIF" and "dorsal bridge plate" groups. Therapy visit number, cost, and payor (insurance type including Medicare, private insurance, worker's compensation, automobile policy, and private pay) were collected.

Results

In total, 1,376 patients met the inclusion criteria. Of these, 713 of the 1,283 (55.6%) patients in the traditional ORIF group and 25 of the 44 patients (56.8%) in the dorsal bridge plate group attended therapy at our institution. Traditional ORIF and dorsal bridge plate patients averaged 12.6(±10) and 24(±18.7) therapy visits in the one-year following ORIF, respectively. Time to last therapy visit was 90.9(±60) and 175.2(±72.1) days in the traditional ORIF and dorsal bridge plate groups, respectively. Total therapy cost was $1,219(±$1,314) and $2,015(±$1,828) in the traditional ORIF and dorsal bridge plate groups with similar out-of-pocket costs.

Conclusions

Dorsal bridge plate fixation patients attended a greater number of therapy sessions, had a longer time from surgery until therapy end, and had a higher therapy total cost relative to traditional ORIF, but both groups had similar patient out-of-pocket therapy costs.

## Introduction

Operative treatment options for distal radius fractures include volar locking plate, fragment-specific plate fixation, dorsal plating, external fixation, and dorsal bridge plate [1]. Volar locking plate fixation is an increasingly popular fixation method [2]. Alternatively, dorsal bridge plate fixation was first reported in 1998 by Burke and Singer as an option for the treatment of distal radius fractures with extensive intra-articular comminution [3]. Dorsal bridge plates are fixed to the dorsal radius proximal to the fracture and to either the second or third metacarpal, which allows for distraction across the radiocarpal joint and prevention of articular fracture displacement. Indications for dorsal bridge plate fixation include comminuted and unstable fractures, fractures with metadiaphyseal extension, and elderly or polytrauma patients who would benefit from immediate weightbearing. The major drawback of the dorsal bridge plate is the need for a second procedure for plate removal [4-7].

Therapy has traditionally been an important part of the recovery from a distal radius fracture with goals to control pain and regain motion, strength, and function [8]. The role of therapy following distal radius fractures is controversial with recent commentary questioning the need for therapy [9-11]. There is heterogeneity in the literature with some reporting improved outcomes with formal therapy relative to home exercise programs [12,13], while others report no difference or improved outcomes with a home exercise program only [14-17]. The rate of therapy utilization varies significantly in the literature from 21% to 80% [14,18,19]. Factors associated with formal therapy utilization were treatment via open reduction and internal fixation (ORIF), Northeast geography, female patients, and middle-aged patients [18].

This study's primary purpose was to compare therapy utilization following the operative treatment of distal radius fractures with traditional ORIF versus dorsal bridge plate fixation. Secondary outcomes were the time to first and last therapy visits and therapy costs.

## Materials and methods

**Figure 1:**
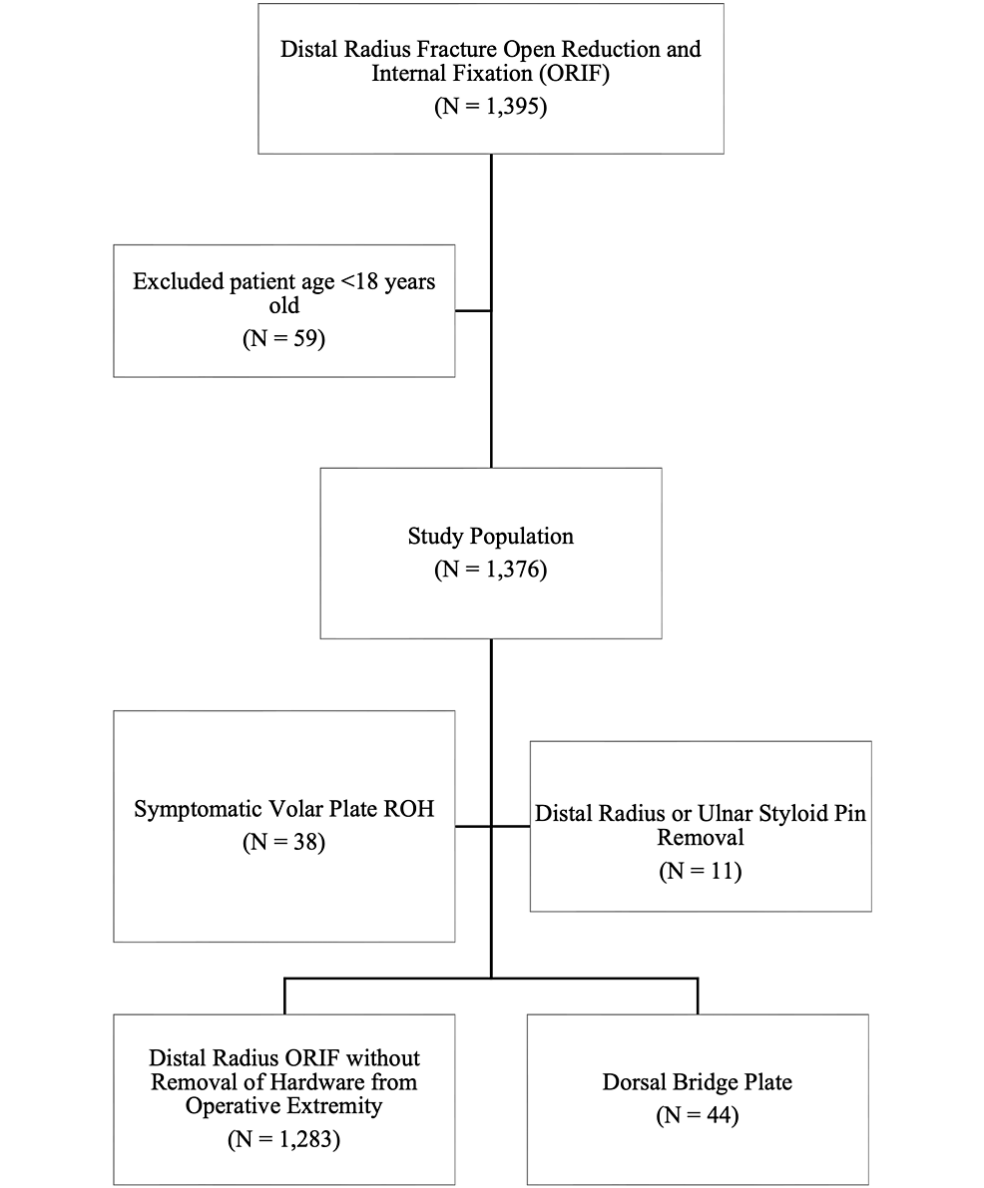
Flowchart of the patient selection process

With approval from the Institutional Review Board of Thomas Jefferson University (approval number: 14D.432), a database search was performed to identify patients who underwent distal radius fracture ORIF as identified by Current Procedural Terminology (CPT) codes 25606, 25607, 25608, and 25609 between January 1, 2021, and August 31, 2022, at Rothman Orthopaedic Institute in Philadelphia, United States. Patients under 18 years old were excluded. A total of 1,376 patients were identified who underwent distal radius fracture fixation during the study period. A second database search was performed for patients who underwent removal of hardware (ROH) as identified by CPT code 20680. Queries were cross-referenced to identify patients who underwent ROH and distal radius fracture ORIF yielding 110 patients. These patients' electronic medical records were retrospectively reviewed to determine the type of ROH procedure performed. A total of 93 patients underwent ROH from the operative extremity. Of these, 38 underwent symptomatic volar plate ROH within one year, and 11 underwent distal radius or ipsilateral ulnar styloid pin ROH and were excluded from the primary analysis. This resulted in two groups: the "traditional ORIF" group who underwent distal radius fracture ORIF without ROH from the operative extremity within one year and the "dorsal bridge plate” group who underwent dorsal bridge plate and subsequent planned ROH (Figure 1).

A therapy database query was performed to identify all physical therapy and occupational therapy visits ("therapy visits") at our orthopaedic specialty practice (our institution) within one year of their index distal radius fracture ORIF. The number and date of therapy visits, total therapy cost, out-of-pocket patient cost, and therapy payor (commercial insurance, Medicare, worker's compensation, or automobile policy) were all collected. The decision to prescribe therapy and the duration of the prescription for therapy were determined by the operative surgeon. For patients in the dorsal bridge plate group who did not receive therapy at our institution, a retrospective review of available outside therapy notes was conducted.

Continuous data are presented as a mean (standard deviation (SD)). Categorical data are presented as cell count (percentage). For demographics between all groups, Kruskal-Wallis or analysis of variance (ANOVA) tests were used to compare continuous data, and chi-squared tests were used to compare categorical data. The two-tailed t-test was used to compare therapy visit number, time to first and last therapy visit, and cost between the dorsal bridge plate and traditional ORIF groups.

## Results

**Figure 2:**
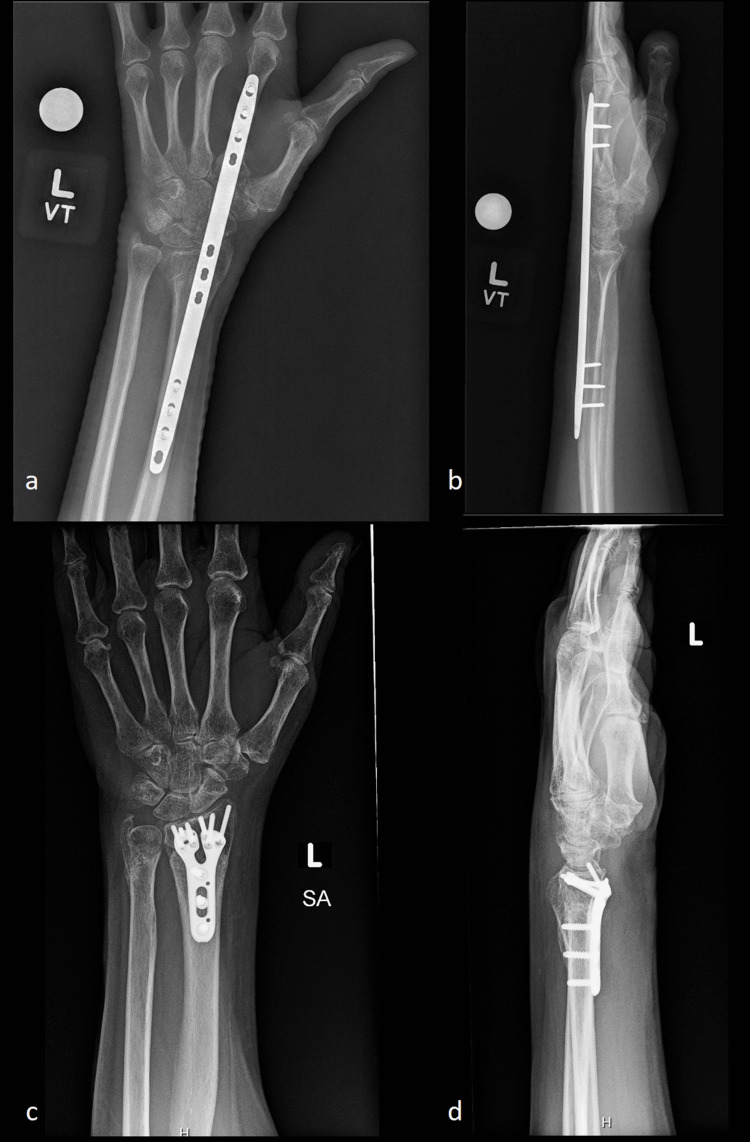
Post-operative distal radius fracture radiographs Post-operative radiographs of dorsal bridge plate fixation (a, b) and volar locking plate fixation (c, d)

During the 20-month study period, 1,376 patients over the age of 18 years old underwent distal radius fracture ORIF. Of these patients, 1,283 were in the traditional ORIF group, and 44 were in the dorsal bridge plate group. Examples of volar locking plate and dorsal bridge plate fixation are shown in Figure 2. Patient demographics are presented in Table 1 and are similar between groups. There were 49 patients who underwent a different ROH procedure from the operative extremity and were excluded from the primary analysis. Patient demographics for these patients are presented in Table 2 and are similar to the included patients (p>0.05). In the dorsal bridge plate group, the average time from dorsal bridge plate ORIF until dorsal bridge plate removal was 109.3 days (SD 51.2).

**Table 1:** Patient demographics ORIF: open reduction and internal fixation; BMI: body mass index

	Traditional ORIF (N=1283)	Dorsal bridge plate (N=44)
Age	59.1 (15.7)	63.3 (15.6)
Sex		
Female	1027 (80%)	32 (72.7%)
Male	256 (20%)	12 (27.3%)
BMI	26.7 (5.73)	27 (5.67)
Current smokers	119 (16%)	3 (12%)
Diabetics	130 (11.9%)	5 (14.3%)
Procedure laterality		
Left	677 (52.8%)	22 (50%)
Right	606 (47.2%)	22 (50%)

**Table 2:** Other removal of hardware patient demographics BMI: body mass index

	Symptomatic volar plate (N=38)	Pin removal (N=11)
Age	59.5 (16.8)	65.3 (15.5)
Sex		
Female	32 (84.2%)	9 (81.8%)
Male	6 (15.8%)	2 (18.2%)
BMI	26.4 (4.52)	27.4 (5.68)
Current smokers	1 (4.35%)	1 (14.3%)
Diabetics	2 (6.06%)	2 (22.2%)
Dementia	2 (6.06%)	2 (22.2%)
Anxiety/depression	2 (6.06%)	2 (22.2%)
Procedure laterality		
Left	13 (34.2%)	4 (36.4%)
Right	25 (65.8%)	7 (63.6%)

In total, 713 of 1,283 patients (55.6%) in the traditional ORIF group and 25 of 44 patients (56.8%) in the dorsal bridge plate group attended therapy at our institution and had therapy data available for analysis. Table 3 summarizes the demographics for this subset of patients. Of patients who underwent therapy at our institution, the traditional ORIF group attended on average 12.6 (SD 10) therapy visits, and the dorsal bridge plate group attended on average 24 (SD 18.7) therapy visits in the one-year period following index distal radius fracture ORIF (p=0.006). Within the dorsal bridge plate group, 56% (14 of 25 patients) attended therapy both before and after ROH, 28% (seven of 25 patients) attended therapy only after ROH, and 16% (four of 25 patients) attended therapy only before ROH (Table 4). Of the dorsal bridge plate group patients who attended therapy both before and after ROH, an average of 40.7% (SD 13%) of visits were attended before the removal of the dorsal bridge plate.

**Table 3:** Patient demographics for patients attending therapy ORIF: open reduction and internal fixation; BMI: body mass index

	Traditional ORIF (N=713)	Dorsal bridge plate (N=25)
Age (years)	59.5 (15.3)	62 (12.5)
Sex		
Female	576 (80.8%)	16 (64%)
Male	137 (19.2%)	9 (36%)
BMI	26.6 (5.6)	27.5 (5.8)
Current smokers	56 (7.9%)	1 (4%)
Diabetics	72 (11.9%)	4 (21.1%)
Procedure laterality		
Left	378 (53%)	14 (56%)
Right	335 (47%)	11 (44%)

**Table 4:** Dorsal bridge plate patients' therapy utilization relative to ROH procedure ROH: removal of hardware

	Therapy before and after ROH (N=14)	Therapy before ROH only (N=4)	Therapy after ROH only (N=7)
Total number of therapy visits	35 (15.1)	5 (4.2)	12.9 (15.3)
Visits before ROH	14.6 (9.3)	5 (4.2)	-
Visits after ROH	20.4 (9.3)	-	12.9 (15.3)
Time from index procedure to first therapy visit (days)	21.2 (7.3)	28.3 (17.5)	151.9 (49.9)
Time from index procedure to last therapy visit (days)	183.34 (37.3)	55 (39.4)	227.7 (63)

Patients started therapy at an average of 26.5 (SD 32.6) and 58.9 (SD 64.8) days post-operatively from their index procedure in the traditional ORIF and dorsal bridge plate groups, respectively (p=0.020). The time from index distal radius fracture ORIF until the last therapy visit was 90.9 days (SD 60) in the traditional ORIF group and 175.2 days (SD 72.1) in the dorsal bridge plate group (p<0.001).

Table 5 summarizes insurance information for these patients. The total cost paid for therapy within the one-year post-operative period was $1,219 (SD $1,314) in the traditional ORIF group and $2,015 (SD $1,828) in the dorsal bridge plate group (p=0.041). Out-of-pocket therapy cost to the patient was zero for 279 of the 713 patients (39.1%) in the traditional ORIF group and 12 of the 25 patients (48%) in the dorsal bridge plate group. Of the patients that had an out-of-pocket cost greater than zero, the average out-of-pocket cost was $285.50 (SD $334.5) in the traditional ORIF group and $232.69 (SD $294.6) in the dorsal bridge plate group (p=0.293).

**Table 5:** Insurance type for patients attending therapy

	Traditional ORIF (N=713)	Dorsal bridge plate (N=25)
Commercial insurance	479 (67.2%)	11 (44%)
Medicare and supplement	161 (22.6%)	10 (40%)
Worker's compensation	38 (5.3%)	4 (16%)
Automobile policy	19 (2.7%)	0 (0%)
Medicare only	13 (1.8%)	0 (0%)
Self-pay only	3 (0.4%)	0 (0%)

Of the 19 dorsal bridge plate patients who did not attend therapy at our institution, limited outside therapy records informing the duration of therapy were available for seven patients. Three patient records reported the duration of therapy as three months (range 2.5-3.5 months), and four patient records reported the total average number of therapy visits as 28.2 (range 18-48 visits). 

Of the 49 patients who underwent a different ROH procedure from the operative extremity and were excluded from the primary analysis, 38 underwent removal of a symptomatic volar plate within one year, and 11 underwent removal of a Kirschner pin. Of these, 68.4% (26 of 38 patients) and 63.6% (seven of 11 patients) of the symptomatic volar plate and pin removal patients, respectively, attended therapy at our institution and had similar demographics to those included in our study (Table 6). These patients attended an average of 20.1 (SD 15.7) and 16.1 (SD 13.2) visits in the symptomatic volar plate and pin removal groups, respectively. In the symptomatic volar plate group, the first and last therapy visits were an average of 53 (SD 58) and 170 (SD 89.1) days after the primary procedure, respectively. In the pin removal group, the first and last therapy visits were an average of 41 (SD 40.2) and 127 (SD 75.4) days after the primary procedure, respectively. The total cost paid for therapy within the one-year post-operative period was $1,819 (SD $1,330) in the symptomatic volar plate group and $1,372 (SD $1,156) in the pin removal group.

**Table 6:** Other removal of hardware patient demographics for patients attending therapy BMI: body mass index

	Symptomatic volar plate (N=26)	Pin removal (N=7)
Age	60.5 (16.8)	70.3 (13.4)
Sex		
Female	2 (7.7%)	0 (0%)
Male	24 (92.3%)	7 (100%)
BMI	26 (4.5)	26.3 (6.3)
Current smokers	1 (3.8%)	0 (0%)
Diabetics	1 (4.3%)	1 (20%)
Procedure laterality		
Left	12 (46.2%)	3 (42.9%)
Right	14 (53.8%)	4 (57.1%)

## Discussion

Patients who underwent dorsal bridge plate fixation attended a greater number of therapy sessions and had a longer time from surgery until the end of therapy completion. The traditional ORIF group attended an average of 12.6 therapy sessions. This aligns with previous reports of therapy session utilization following distal radius fracture ranging from six to 16 sessions [13,14,19,20]. These studies report on non-operatively treated distal radius fractures and traditional ORIF, but none of these studies included patients treated with dorsal bridge plate fixation. Here, patients in the dorsal bridge plate group attended nearly twice as many therapy sessions (24 sessions versus 12.6 sessions) as the traditional ORIF group. These patients also attended therapy for a longer time from their index ORIF, averaging 175.2 days from the index ORIF procedure relative to 90.9 days in the traditional ORIF group. The reason for the higher number of therapy visits and the longer time from procedure to completion of therapy is multifactorial, including the need for an additional procedure, the period of wrist immobilization associated with dorsal bridge plate fixation, and pre-operative patient and fracture characteristics that may have influenced the choice of fixation method. 

Patients are indicated for dorsal bridge plate due to either patient factors or the complexity of the fracture pattern. Patient factors that favor dorsal bridge plate fixation include polytrauma patients and elderly patients due to the need for immediate weightbearing through the operative extremity. Fracture patterns that favor dorsal bridge plate fixation include complex, severely comminuted, unstable distal radius fractures and fractures with metadiaphyseal extension [6,7]. We demonstrate similar patient demographics between groups as presented in Table 1 and Table 3, but additional patient and fracture differences between groups may account for greater therapy utilization. For example, it is postulated that patients who underwent dorsal bridge plate fixation may have had different baseline activity demands and functional status and presented with more comminuted, displaced fractures that could have impacted their therapy needs. This retrospective study was not designed to identify these factors, and further studies are necessary to better understand the impact of these variables on therapy utilization. Alternatively, the increased therapy utilization may reflect the need for a second procedure to remove the dorsal bridge plate or increased stiffness from a prolonged period of wrist immobilization [21]. Though patients in the dorsal bridge plate group started therapy later relative to the traditional ORIF group (26.5 days post-operatively versus 59.8 days post-operatively, respectively), just over half of patients in the dorsal bridge plate group attended therapy both before and after ROH. These patients attended 40.7% of their therapy visits prior to ROH and had more therapy (average of 35 visits) than dorsal bridge plate patients who only attended therapy before (five visits) or after (12.9 visits) ROH.

The greater therapy utilization in the dorsal bridge plate group corresponds with a higher therapy total cost, but similar patient out-of-pocket therapy costs in both groups. Total therapy cost averaged $2,015 in the dorsal bridge plate group relative to $1,219 in the traditional ORIF group. Therapy costs represent only a portion of the total cost of treatment for distal radius fractures. Shauver et al. report that Medicare spent over 170 million dollars on distal radius fracture attributable expenses in 2007 and project this value to increase with time [22]. An insurance database study by Shah et al. analyzing data from 2007 to 2015 reported that the cost averaged $550-650 per therapy visit for distal radius fractures [19]. Here, we report a higher cost of therapy, but this is likely attributed to two main factors. First, the increased timeframe of data collection of one year versus 90 days post-operatively allows us to capture patients who attended a longer period of therapy. Second, geographic price differences may affect cost as our study was conducted in the Northeast, but 70% of patients in the study by Shah et al. are from the South [19]. Though therapy contributes to treatment costs, the highest contributors to cost in operatively treated distal radius fractures are the implants used, with higher costs associated with different manufacturers and the number of implants used [23,24]. These studies do not reference the cost of dorsal bridge plates, and further studies are necessary to understand the total cost difference in utilizing dorsal bridge plate fixation, including differences in cost for a second procedure, implant cost, and office visits. This study suggests that the increased cost of therapy in the dorsal bridge plate group is another factor that should be considered in future cost analyses for distal radius fracture treatment. 

Dorsal bridge plate fixation is limited by the need for a planned second procedure for hardware removal. There were 49 additional patients who required hardware removal from their operative extremity during the study period, of which 77.6% underwent removal of the symptomatic volar plate and 22.4% underwent Kirschner wire removal. Interestingly, patients who had symptomatic volar plate removal had a similar increase in the total number of therapy sessions compared with dorsal plate removal (average 20.1 and 24 sessions, respectively). This increase was not as high in the pin removal group (16.1 sessions). Together, this may suggest that the increase in therapy utilization may be a reflection of multiple procedures as opposed to a reflection of increased stiffness associated with fixation crossing the radiocarpal joint, though further studies are necessary to evaluate this hypothesis.

This study is limited to patients who attended therapy within our regional orthopaedic practice, which only represents 55.8% of our patients. To better understand this limitation, we performed a limited retrospective review of available outside therapy records for seven patients in the dorsal bridge plate group. This demonstrated an average duration of therapy of three months for the three patients with a reported duration of therapy and an average of 28.2 therapy visits for the four patients with a recorded number of therapy visits. Therapy utilization for this subset of patients is similar to our study cohort. Patients who did not attend therapy within our institution either attended therapy through an outside institution or did not attend therapy at all. Further studies of therapy utilization at a physical and occupational therapy office not associated with an orthopaedic practice are needed to better understand the generalizability of our findings.

## Conclusions

There is greater therapy utilization following distal radius fractures in patients treated with dorsal bridge plating in comparison to traditional ORIF. This includes both a greater number of therapy visits and a longer duration of therapy from the time of index distal radius fracture ORIF. The decision-making behind the technique utilized for the operative treatment of a distal radius fracture is multifactorial. While the findings of this study are unlikely to alter this decision, these findings can help surgeons better counsel their patients on the expected duration and cost of post-operative therapy visits following treatment with dorsal bridge plating versus traditional ORIF.
